# Microplate-based platform for combined chromatin and DNA methylation immunoprecipitation assays

**DOI:** 10.1186/1471-2199-12-49

**Published:** 2011-11-18

**Authors:** Jingjing Yu, Qinghua Feng, Yusong Ruan, Radko Komers, Nancy Kiviat, Karol Bomsztyk

**Affiliations:** 1 UW Medicine Lake Union, University of Washington, Seattle, WA 98109, USA; 2 Department of Medicine, Oregon Health and Science University, Portland, OR 97201, USA

## Abstract

**Background:**

The processes that compose expression of a given gene are far more complex than previously thought presenting unprecedented conceptual and mechanistic challenges that require development of new tools. Chromatin structure, which is regulated by DNA methylation and histone modification, is at the center of gene regulation. Immunoprecipitations of chromatin (ChIP) and methylated DNA (MeDIP) represent a major achievement in this area that allow researchers to probe chromatin modifications as well as specific protein-DNA interactions *in vivo *and to estimate the density of proteins at specific sites genome-wide. Although a critical component of chromatin structure, DNA methylation has often been studied independently of other chromatin events and transcription.

**Results:**

To allow simultaneous measurements of DNA methylation with other genomic processes, we developed and validated a simple and easy-to-use high throughput microplate-based platform for analysis of DNA methylation. Compared to the traditional beads-based MeDIP the microplate MeDIP was more sensitive and had lower non-specific binding. We integrated the MeDIP method with a microplate ChIP assay which allows measurements of both DNA methylation and histone marks at the same time, Matrix ChIP-MeDIP platform. We illustrated several applications of this platform to relate DNA methylation, with chromatin and transcription events at selected genes in cultured cells, human cancer and in a model of diabetic kidney disease.

**Conclusion:**

The high throughput capacity of Matrix ChIP-MeDIP to profile tens and potentially hundreds of different genomic events at the same time as DNA methylation represents a powerful platform to explore complex genomic mechanism at selected genes in cultured cells and in whole tissues. In this regard, Matrix ChIP-MeDIP should be useful to complement genome-wide studies where the rich chromatin and transcription database resources provide fruitful foundation to pursue mechanistic, functional and diagnostic information at genes of interest in health and disease.

## Background

The study of chromatin biology has emerged as a new paradigm to understand the pathophysiology of critical events responsible for diseases such as cancer [1-3], diabetes [4,5], cardiomyopathies [6], ageing [7] and many others. Chromatin is a compact, but remarkably dynamic, structure that plays a critical role in transcription, DNA replication and repair [8,9]. Its structure and function is regulated through covalent modifications of DNA and nucleosome histones as well as substitution by a variety of histone variants.

Mammalian DNA can be modified by cytosine methylation which involves the addition of a methyl group to the 5 position of a cytosine (5mC) generally, but not always [10], preceding guanosine in the DNA sequence (cytosine-phosphate-guanine, CpG). Mammalian DNA methylation is mediated by DNA methyltransferases (DNMTs) including DNMT1 (responsible for maintaining proper methylation levels during replication and possibly DNA repair), DNMT3a and DNMT3b (responsible for *de novo *methylation during embryogenesis) [11]. CpG islands are genomic regions that contain a high frequency of CpG sites typically > 300 base pairs in length. Most studies have focused on methylation of CpG islands in the gene's promoter region where it is associated with gene repression [12]. This is thought to result from a blockade of transcription factor binding to genomic targets [13]. Although methylation of CpG islands nested within transcribed regions has not been extensively studied, new evidence suggests that the role of DNA methylation in transcription elongation and termination is just as important as CpG methylation in the regulation of transcription initiation [14-16]. Because of its importance in biology of disease several different methods were developed to assay DNA methylation. Bisulfite sequencing, bisulfite conversion-based MethyLight, methylation-sensitive digestion, and methyl-DNA immunoprecipitation (MeDIP) are some of the well established methods to assay DNA methylation both at specific sites and genome-wide [10,17-20]. Because of its simplicity and low cost MeDIP is increasingly becoming a popular method [21].

Histone post-translational modifications (PTMs; e.g., include acetylation, methylation, and phosphorylation) are the major avenues that regulate chromatin dynamics: they expose, or close, docking sites for a host of other molecules, including chromatin remodeling and transcription factors [9,22,23]. To date, more than 100 different histone amino acid residues have been shown to be modified [9,24,25]. A host of enzymes that modify specific histone amino acid residues have been identified [8,9,24]. These include, but are not limited to, histone methyltransferases [9], demethylases [26], acetyltransferases [27], deacetylases [28], kinases [29,30] and phosphatases [31]. Many, if not most of these enzymes, are directly recruited to specific genomic regions, for example, very recently kinases [32-36] and phosphatases [31,36-38] were discovered to be directly recruited to their target genes. The significant progress in this area of research was facilitated by the introduction of the chromatin immunoprecipitation (ChIP) assay [39-41].

Although chromatin studies are providing compelling evidence for dynamic interchange between histones and DNA methylation [42], typically DNA methylation and histone modification studies have been done independently of each other and most often by different laboratories using low throughput technologies. Here, we describe a simple and easy-to-use microplate-based platform for combined analysis of DNA methylation, histone modifications and chromatin-bound enzymes, Matrix ChIP-MeDIP.

## Methods

### Reagents

Bovine serum albumin (BSA), phosphate buffered saline (PBS), salmon sperm DNA, transfer RNA (tRNA), 5-aza-2'-deoxycytidine (DAC), trichostatin A (TSA), and protein A were from Sigma, and proteinase K was from Invitrogen. Matrix ChIP-MeDIP 96-well polypropylene plates were from Bioexpress. Formaldehyde, ethanol, NaCl, EDTA, Triton X-100, NP-40, Tris-HCl, leupeptin, PMSF, p-nitrophenyl phosphate, NaF, Na_3_VO_4_, Na_2_MoO_4 _and β-glycerophosphate were from Sigma. Dulbecco's Modified Eagle Medium (DMEM), McCoy's medium, penicillin/streptomycin (P/S), Glutamax, fetal bovin serum (FBS), trypsin/EDTA were obtained from Invitrogen. The antibodies were commercially available as listed in Table 1.

**Table 1:** Antibodies

Antibody	**Catalog No**.	Source	Manufacturer	Peptide immunogen
Flag M2	F 1804	Murine monoclonal	Sigma	Recognize a FLAG peptide sequence at the N-terminus, Met-N-terminus, C-terminus, or internal sites of a fusion protein
H2A.Z	39113	Rabbit polyclonal	Active Motif	C-terminus of human histone H2A.Z
H3K9/14Ac	06-599	Rabbit polyclonal	Millipore	KLH-conjugated peptide corresponding to amino acids 1-20 of Tetrahymena histone H3 (ARTKQTAR[K*]STGG[K*]APRKQLC) where K* is acetylated.
H3K27m3	Ab6002	Mouse monoclonal	Abcam	Synthetic peptide derived from residues 1-100 of human histone H3, trimethylated at K27
mCpG	AMM99021	Mouse monoclonal	Aviva	5-methylcytosine conjugated to ovalbumin
mCpG	MAb-5MECYT	Mouse monoclonal	Diagenode	Raised against 5-methyl Cytidine conjugated to ovalbumine
Pol II CTD (4H8)	Sc-47701	Mouse monoclonal	Santa Cruz	C-terminal domain peptide

### Cell Lines and Treatment

HeLa cells were obtained from the American Type Culture Collection (ATCC, Manassas, VA), and grown in DMEM supplemented with P/S (0.5 units/ml), Glutamax (2 mmol/l), and 10% (v/v) FBS.

For inhibitor studies, HeLa cells were seeded in T150 tissue culture flasks at a low density and treated with 200 nmol/l of DAC for 3 days and 300 nmol/l of TSA for the last 24 hours. Media containing fresh drugs was replaced daily. Controls consisted of cells treated identically with drug solvents (PBS and 100% ethanol), and all experiments were done in triplicate. After treatment, cells were removed from flasks using trypsin/EDTA solution and rinsed with PBS. Cell pellets were ready for chromatin or DNA isolation.

### Human cervical tissues

Ten frozen cervical tissue samples (5 with normal histology and 5 with invasive cervical cancer) were selected from our established IRB-approved specimen repository at University of Washington. These samples were collected from our previous studies conducted in Senegal investigating the relationship between cervical neoplasia, HIV infection, and inflammation [43].

### Mice

Two-month-old male FVB and OVE26 mice on FVB background [44] and two-month- old C57BL/6 and C57BL/6 ob/ob [36] mice were purchased from The Jackson Laboratory (Bar Harbor ME and were maintained and sacrificed as described previously [45]. Blood was collected just prior to sacrifice. Subsequently, kidneys were removed and flash frozen in liquid nitrogen. Blood glucose was measured using One Touch Ultra system (LifeScan Inc.). All procedures were done in accordance with current NIH guidelines and approved by the Animal Care and Use Committee of the University of Washington.

### Chromatin and DNA preparation

For ChIP assays, cell pellets (10^6 ^cells) or tissues (10-20 mg) were cross-linked with formaldehyde final concentration 1.42% for 15 minutes at room temperature and then formaldehyde was quenched with 125 mM glycine for 5 minutes at room temperature as previously described [46]. Cross-linked cells/tissues were lysed using IP buffer [150 mM NaCl, 5 mM EDTA, 1% Triton X-100, 0.5% NP-40, 50 mM Tris-HCl (pH 7.5)] containing 10 μg/μl leupeptin, 0.5 mM PMSF, 30 mM p-nitrophenyl phosphate, 10 mM NaF, 0.1 mM Na_3_VO_4_, 0.1 mM Na_2_MoO_4, _and 10 mM β-glycerophosphate [47,48]. Chromatin was sheared using Diagenode Bioruptor (100 µl IP buffer, 30 rounds 30 seconds ON/30 seconds OFF, high power, 4°C). The suspension was cleared by centrifugation at 12,000 g (10 minutes at 4°C), and the supernatant, representing sheared chromatin, was aliquoted and stored at -80°C. DNA input was prepared by incubation sheared chromatin with proteinase K (0.2 μg/μl) for 15 minutes at 55°C, followed by 15 minutes at 95-100°C in a PCR cycler.

For MeDIP, single stranded DNA (ssDNA) was isolated from HeLa cell pellets, and tissues using Omega Bio-Tek Tissue DNA Kit (Norcross, GA), followed by ultrasound-shearing and denaturation (boiling 10 minutes). We found that using the proteinase K method (as above) for DNA purification from uncross-linked and cross-linked sheared chromatin gave the same MeDIP results. Thus, the same cross-linked sheared sample can be used for input chromatin in ChIP and for purification of ssDNA as input for MeDIP assays. For MeDIP, purified DNA was treated with 1 ng/μl RNase.

### Microplate-based immunoprecipitation (Figure 1)

**Figure 1:**
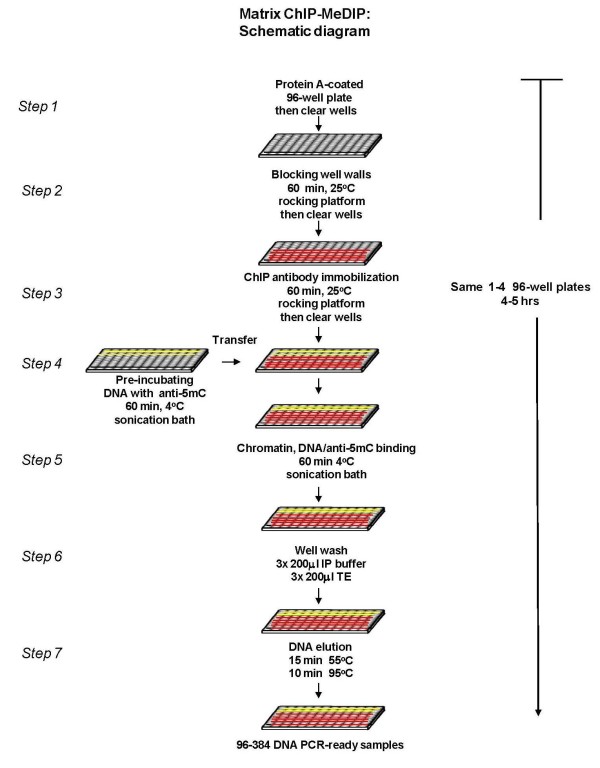
**Diagram of the microplate-based Matrix ChIP-MeDIP method**. Batches of polypropylene plates are coated overnight with protein A (room temperature) and are then stored sealed up to 2 weeks (4°C) and used when needed. Starting with protein A-coated plates the ChIP-MeDIP assay takes 4-5 hours to generate PCR-ready DNA. Detailed protocol is described in "Method" section. The red and yellow lines exemplify rows of wells with ChIP and MeDIP antibodies, respectively.

#### Step1. Protein A-coated microplate preparation

UV-irradiated 96-well plates were coated with protein A by incubating overnight at room temperature with 20-100 μl of 2 μg/μl protein A in PBS per well. Parafilm-sealed plates were stored up to 2 weeks (4°C) without loss of activity. The same microplate(s) was used for ChIP and MeDIP assays- typically 1-3 rows of wells were used for MeDIP and the rest for ChIP.

#### Step2. Blocking well walls

Prior to use, plates were washed twice with 200 μl of PBS and well walls were blocked with 200 μl of blocking buffer for 60 minutes at room temperature. The wells used for MeDIP were blocked with IP buffer/5% BSA (MeDIP blocking buffer) and those for ChIP with IP buffer/5% BSA containing 100 μg/ml sheared salmon sperm DNA (ChIP blocking buffer). In all steps, vacuum aspirator wand was used to clear wells of buffers.

#### Step3. Attachment of ChIP antibodies to well walls

ChIP wells were cleared of blocking buffer and then ChIP antibodies diluted in ChIP blocking buffer were incubated in these "ChIP" wells for 60 minutes at room temperature. Wells were cleared and then appropriate amount of sheared chromatin diluted in ChIP blocking buffer was added to "ChIP" wells.

#### Step4. Pre-incubation of 5mC antibody with denatured ssDNA

Sheared ssDNA (0.1-1 ng DNA/μl) was pre-incubated (either plain tubes or 96-well plates) in MeDIP blocking buffer with anti-5mC antibody in an ultrasonic water bath (Branson 3510) for 60 minutes at 4°C. And then the mixture was transferred to blocked "MeDIP" wells. The reaction volume varied depending on the number of genes to be studied. *Steps 3-4 *were done at the same time.

#### Step5. Capture of methylated ssDNA and chromatin to well walls

Microplate(s) were covered with a sealing film and capture of chromatin and DNA complexes to well walls was done by floating the microplates in ultrasonic water bath (Branson 3510) for 60 minutes at 4°C.

#### Step6. Well washes

Wells were washed three times with ice-cold IP buffer followed by three washes with ice-cold TE buffer (pH 7.0).

#### Step7. Elution of PCR-ready DNA

After washes, appropriate amount of elution buffer (25 mM Tris, pH 10, 1 mM EDTA, and 0.2 μg/μl proteinase K containing 1% IP buffer) was added to each well. Input DNA was prepared by adding the same amount of chromatin/DNA to wells containing elution buffer on the same plate as the ChIP-MeDIP samples. The plates were then sealed with PCR sealing film. PCR-ready DNA was eluted/purified in a 96-well PCR cycler applying one cycle of heating (55°C for 15 minutes) followed by one cycle to reverse cross-linking and to inactivate proteinase K (95°C for 15 minutes).

### Beads MeDIP

Immunoprecipitation of methylated DNA using beads was done using an adaptation of the Fast ChIP method [46]. Briefly, sheared ssDNA was pre-incubated in MeDIP blocking buffer (30 μl) with anti-5mC antibody in either 96-well plates or plain tubes in an ultrasonic water bath (Branson 3510) for 60 minutes at 4°C. The mixture was then transferred to fresh tubes containing 10 μl of washed protein A agarose beads (Pharmacia). The slurry was rotated for 30 minutes (4°C) and then the beads were washed three times with 1 ml of cold IP buffer and three times with 1 ml of cold TE buffer containing no inhibitors. Then 60 μl of 10% Chelex/Proteinase K (100 μg/ml) was added to the beads and the slurry was incubated for 30 minutes at 55°C while shaking, followed by another round of boiling for 10 minutes. Suspension was centrifuged and supernatant was collected. Eluate was used directly as a template in PCR analysis.

### PCR analysis

1-2 μl of eluted DNA was used in 2-4 μl real-time PCR reactions (ABI7900HT). All PCR reactions were run in triplicate. PCR primers were designed using the Primer3 software (http://frodo.wi.mit.edu/) and shown in Table 2. PCR calibration curves were generated for each primer pair from a dilution series of sheared total human or mouse genomic DNA. The PCR primer efficiency curve was fit to cycle threshold (Ct) versus log (genomic DNA dilutions) using an r-squared best fit. DNA concentration values for each ChIP-MeDIP and input DNA samples were calculated from their respective average Ct values. Levels of 5mC, Pol II, enzymes and histone marks in immunoprecipitated samples were expressed as a copy number ratio of sample immunoprecipitated with specific antibody to that of input DNA (% Input) [47].

**Table 2:** PCR primers.

Species	Gene	Purpose	Forward	Reverse	Probe
Human	*ALU*	Real-time PCR	CGGTGGCTCACGCCTGTA	GAGTGCAGTGGCGCGATC	
Human	*ALU*	MethyLight	GGTTAGGTATAGTGGTTTATATTTGTAATTTTAGTA	ATTAACTAAACTAATCTTAAACTCCTAACCTCA	CCTACCTTAACCTCCC
Human	*LINE*	Real-time PCR	ACGGAATCTCGCTGATTGCT	CGTTGCCGCCTTGCA	
Mouse	*MCP-1*	Real-time PCR	GAATGAAGGTGGCTGCTATG	AACCCAGAAACATCCAATTCTC	
Human	*SFRP1*	Real-time PCR	CTTACCTTGGGGCTTGGAG	CTACTGGCCCGAGATGCTTA	TTTCGCGTTTTTTTGT
Human	*SPARC*	Real-time PCR	GGTTTCCTGTTGCCTGTCTC	GGGGGTCACACATACCTCAG	
Human	*SPARC*	MethyLight	TTTCGCGGTTTTTTAGATTGTTC	CATACCTCAATAACAAACAAACAAACG	CAAAACGCGCTCTC
Mouse	*TGF-β1*	Real-time PCR	TTTGAGACTTTTCCGCTGCT	AATAGGGGCGTCTGAGGAAC	

### MethyLight assay

MethylLight assays were done using established protocols [19]. Briefly, CpG island sequences were identified using the University of California Santa Cruz genome browser (http://genome.ucsc.edu/) or from genomic information using CpGPlot (http://www.ebi.ac.uk/emboss/cpgplot/). MethyLight primers and MGB probes were designed using Primer Express (ABI). Sequences of primers and probes are listed in Table 2. Cervical tissue DNA was converted using EpiTect Bisulfite Kit (Qiagen). For MethyLight, 1 µl of bisulfite-converted DNA solution was combined with 600 nmol/l of each primer and 200 nmol/l of probe in ABI Universal PCR Master Mix and amplified under the default cycling conditions for Prism HT7900 instrument. Human sperm DNA and human sperm DNA methylated *in vitro *using the SssI (CpG) methylase (New England Biolabs, Beverly, MA), respectively, were used as U (unmethylated) and M (fully methylated) control DNA. Copy number of specific genes in samples was obtained using the standard curve generated from known amount of bisulfite-converted M sequence. The "percent of methylated reference" (PMR) values were calculated using a formula: ([geneX mean value for the sample]/[ALU mean value for the sample])/([geneX mean value for the M.SssI reference]/[ALU mean value for the M.SssI reference])*100% [49].

## Results and Discussion

MeDIP is typically done in test tubes with anti-5mC antibody immobilized to beads employing either centrifugation or a magnet [18]. Our goal was to develop a simple and low cost high-throughput microplate-based MeDIP method that could be used in combination with chromatin immunoprecipitation [47].

### Microplate-based MeDIP method development

The standard MeDIP protocol consists of several steps. i) isolation of genomic DNA. ii) DNA fragmentation. iii) DNA denaturation to generate single-stranded DNA. iv) immunoprecipitation of methylated DNA fragments using an antibody to 5mC. v) detection of specific sequence(s) by PCR or other methods. The abundant *ALU *and *LINE *repetitive elements are heavily methylated and have been used as surrogates to assess global DNA methylation [50]. *SFRP1 *gene is also methylated [51] and was used as another test gene. Treatment of cells with DNA methylation inhibitor DAC has previously been shown to decrease methylation of both *ALU *and *LINE *elements [43]. We used cervical carcinoma HeLa cells treated with or without methylation inhibitors and tested these genes as readouts to develop a microplate-based MeDIP protocol.

The most critical step was to develop a high efficiency specific immunocapture of methylated DNA fragments to well walls while maintaining low background binding. To do that we used 96-well protein A-coated polypropylene plates that have lower background binding than polystyrene plates. To minimize non-specific binding in microplate ChIP assay we blocked the well walls with 5% BSA and sheared salmon sperm DNA in IP buffer [47]. Given that salmon sperm DNA is also methylated, it could not be used for blocking the well walls. Thus, we tested several blocking media: BSA, BSA combined with tRNA or microccocal DNA. We found that BSA alone was as good blocker of non-specific binding of sheared denatured DNA as having it combined with either tRNA or microccocal DNA. Thus, in MeDIP we used 5% BSA in IP buffer as a blocking means to minimize non-specific binding of DNA.

**Figure 2:**
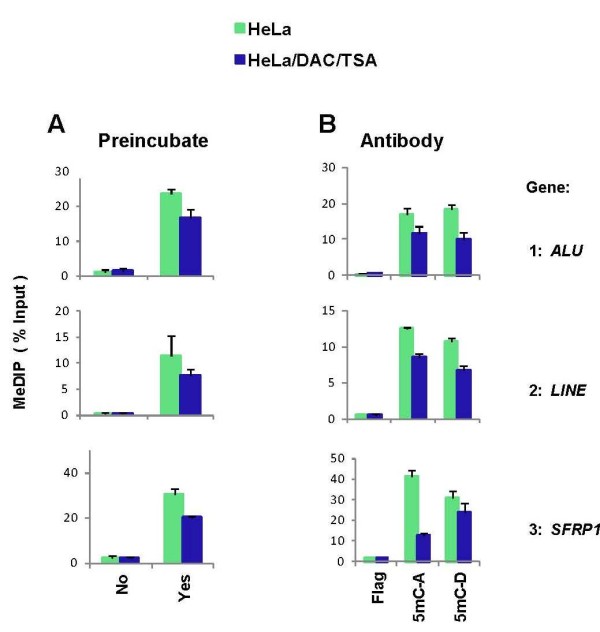
**Optimization and testing the microplate-based MeDIP**. HeLa cells were treated with 200 nM of DAC for 3 days and 300 nM of TSA for the last 24 hours. Media containing fresh drugs was replaced daily. Control cells (vehicle) treated identically with drug solvents (PBS and 100% ethanol). Cells were trypsinized and washed with PBS. For MeDIP input DNA was deproteinized using proteinase K, sheared by ultrasound (Bioruptor), RNase treated and denatured. Assays were done using in-lab made protein A-coated polypropylene 96-well plates. ***A, ***MeDIP was tested either when anti-5mC antibody (Diagenode) was first attached to wells and then DNA was added (*No*) or the DNA was preincubated (*Yes*) with the antibody first and then the mixture was added to protein A-coated 96-well plates and binding was done in ultrasonic bath. After the binding step, wells were washed, DNA was eluted and used in real-time PCR using primers to *ALU *and *LINE *elements as wells as the *SFRP1 *gene. ***B*, **MeDIP was done with the preincubation step using monoclonal anti-5mC antibodies from either Diagenode (5mC-D) or Aviva (5mC-A) companies. Monoclonal anti-Flag tag antibody was used as control. Results are expressed as % Input, mean ± SEM of three independent biological replicates.

In microplate-based ChIP assay antibodies are first attached to protein A-coated well walls, then sheared chromatin in blocking buffer is added to wells and chromatin immunocapture is carried out using low energy ultrasound [47]. We found that with this approach immunocapture efficiency of methylated DNA using anti-5mC antibody was low. In bead-based ChIP assay the immunocapture is more efficient when the chromatin is first pre-incubated with antibody and then the mixture is added to the beads [46]. Thus, we compared immunocapture efficiency when either the DNA was added to wells coated with protein A and anti-5mC antibody without pre-incubation or when the DNA was first pre-incubated in ultrasonic bath with the anti-5mC antibody and then the mixture was added to protein A-coated wells. Binding was done with 96-well plates floating in ultrasonic bath to facilitate antibody-antigen binding [46]. After washes, DNA was eluted from the well walls and analyzed in real-time PCR using primers to *ALU *and *LINE *elements as well as *SFRP1*. As shown in Figure 2A, pre-incubation with the anti-5mC antibody increased the efficiency of immunocapture by 10-20 folds. These results also show that the level of DNA pull-down from HeLa cells treated with DNA methylation inhibitor was lower compared to untreated cells, providing evidence for anti-5mC antibody specificity. The modest DAC-induced decrease in DNA-methylation is similar in magnitude to that reported in other cell lines using bisulfate PCR [50].

To further confirm specificity of the pull-down we compared DNA immunocapture using different monoclonal anti-5mC antibodies from two vendors, Diagenode and Aviva. Monoclonal Flag antibody was used as the mock control. Figure 2B shows that the level of immunocapture with Diagenode and Aviva anti-5mC antibodies were similar, and the specific signal was 10-20 times greater than that with the mock Flag antibody. Taken together these results indicate that the microplate-based procedure allows specific immunocapture of methylated DNA.

**Figure 3:**
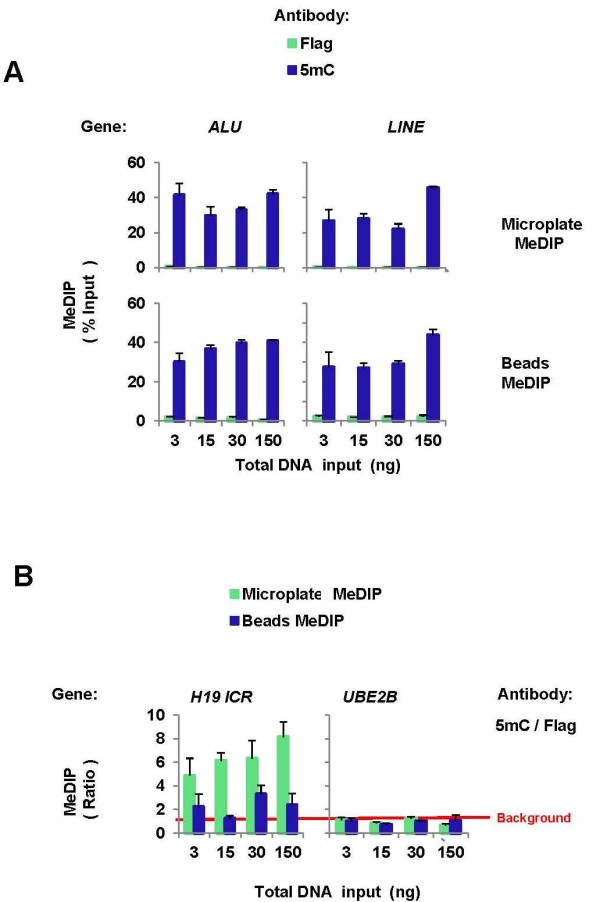
**Validation of microplate-based MeDIP assay**. ***A*, **Denatured HeLa cell DNA was preincubated with either monoclonal anti-5mC or Flag (mock control) monoclonal antibody (0.5 μg each) in binding buffer (60 μl, 60 minutes at 4°C) in ultrasonic bath. After antibody preincubation, the mixture was divided into two 30 μl aliquots, one was used in microplate MeDIP (*Microplate MeDIP*) and the other in protein A-agarose beads MeDIP (*Beads MeDIP*). For each step the same buffer volumes were used in microplate- and beads-based assays. The X-axis shows total DNA input used per IP (nanograms DNA/30 μl IP reaction). After binding, wells and beads were washed with IP buffer and TE buffer. DNA was purified from well walls using 60 μl elution buffer/proteinase K and from beads using 60 μl 10% Chelex/proteinase K [46]. Eluted DNA was used in real-time PCR using primers to *ALU *and *LINE *elements. Results are expressed as % Input, mean ± SEM of three independent biological replicates. ***B*, **As in (A) microplate and beads immunoprecipitated DNA was used in real-time PCR using primers to either the methylated H19 imprinted control region (*H19 ICR*) or the unmethylated promoter region of the house keeping gene *UBE2B *[18]. Data are expressed as ratio of the 5mC signal to the Flag mock (*5mC/Flag*). The red horizontal line represents background binding, 5mC/Flag ratio = 1.

Next we compared performance of the microplate and beads MeDIP assays (Figure 3). A range of HeLa cell total DNA input were pre-incubated with either anti-5mC or Flag (mock) antibody as above in ultrasonic bath. After binding, equal aliquots of the antibody-DNA mixture were incubated either with suspension of protein A beads or added to the protein A-coated microplate wells. Beads and wells were washed with same buffers and DNA was purified from the beads with Chelex [46] and from the well walls with elution buffer as above [47]. Proteinase K was used in both methods as before [46,47]. Purified DNA was analyzed in real-time PCR using primers to *ALU, LINE *elements as well as the known methylated H19 imprinted control region (*H19 ICR*) or the unmethylated promoter region of the housekeeping gene *UBE2B *[18]. Comparison of the microplate and beads MeDIP at the highly abundant *ALU *and *LINE *elements loci showed similar efficiency of immunoprecipitation with the two methods, approximately 20-40% of input. The level of immunoprecipitation was approximately the same for the input DNA range tested (3-150 ng of DNA in 30 μl reaction volume). Although the non-specific binding was higher with the beads method, the specific binding was similarly high with the two methods due to abundance of the heavily methylated *ALU *and *LINE*. When tested on the single copy imprinted *H19 ICR *locus, the performance of the microplate MeDIP was superior to the beads method. To more quantitatively compare the specific and non-specific binding the 5mC immunocapture signal was divided by the Flag signal [46]. In the range of DNA input tested the specific signal measured with the beads was approximately 2-fold over the background compared to 5-8-fold for the microplate. The difference in performance between the two methods was more pronounced at the low level of DNA input (3 ng DNA) where the specific 5mC signal was not different from Flag background using beads, but 4-5 folds higher using the microplate. With either method, at the unmethylated *UBE2B *site the 5mC signal was not different from the Flag background. We used 1 μl of the MeDIP DNA template per real-time PCR reaction which was done in triplicates. Thus, at the lowest DNA amount input (3 ng) the microplate MeDIP method was sufficiently sensitive to test methylation at nine different genomic sites (3 ng in 30 μl volume/well for 5mC and Flag each, total 9 ng DNA amount including input DNA equivalent to ~1500 cells, or ~150 cells/one genomic site).

The schematic diagram of the combined ChIP-MeDIP microplate method is illustrated in Figure 1. Starting with chromatin one individual can generate 96-384 PCR-ready DNA samples in 4-5 hours (1-4 of 96-well plates).

### Application of Matrix ChIP-MeDIP to clinical cancer samples: Combined ChIP-MeDIP demarcates cancer from normal tissue better than MeDIP alone

Altered Pol II transcription and chromatin structure is one of the critical hallmarks of cancer [52], a feature that has been heavily exploited to search for tumor biomarkers that could have diagnostic, prognostic and therapeutic applications. Until now, these studies have largely relied on using either DNA methylation or histone marks but not both. Given the fact that the cancer phenotype reflects extensive alterations in the chromatin structure the capability to integrate Pol II profile, DNA methylation and histones information at cancer-critical loci will not only advance our understanding of transcription/chromatin biology of cancer but should also yield better tumor biomarker.

**Figure 4:**
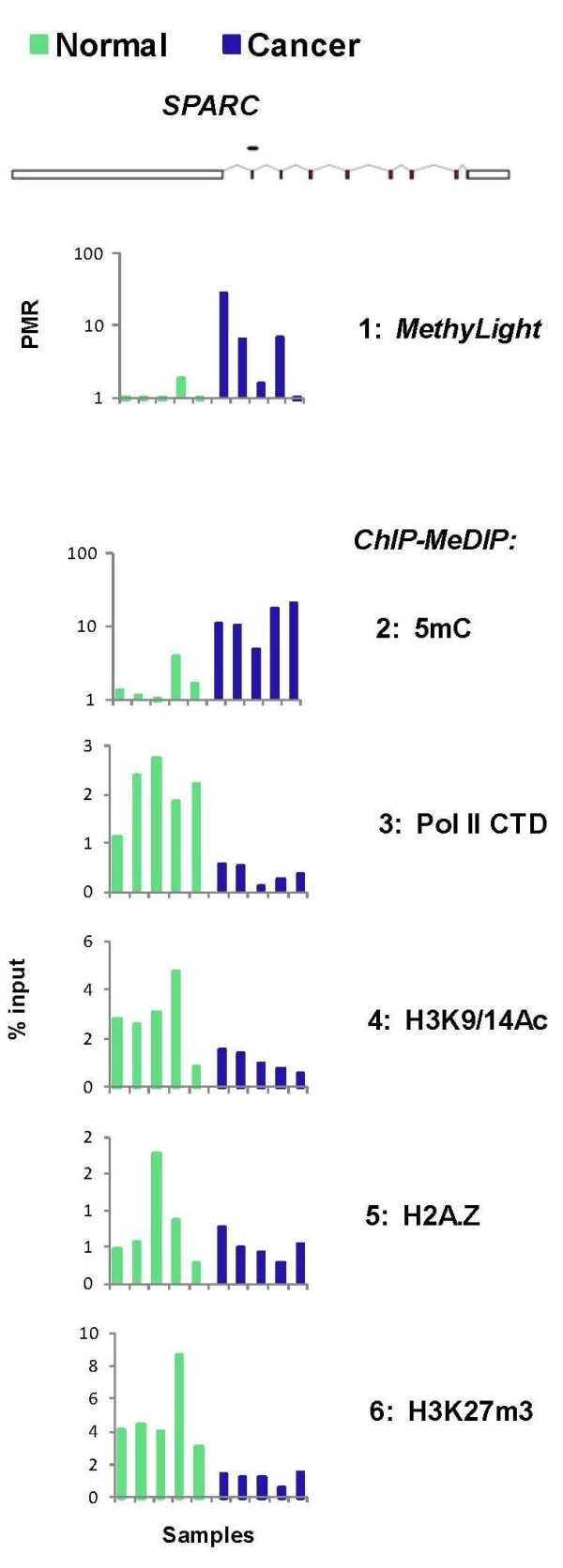
**Comparison of MethyLight and Matrix ChIP-MeDIP assay of normal cervix tissue and cervical cancer**. *MethyLight*, DNA purified from clinical cancer and normal tissues was bisulfite-converted and amplified using *SPARC *gene primer in Taqman PCR [43]. Methylight results are expressed as PMR values for each individual sample. *ChIP-MeDIP*, frozen (-80°C) archived cervical tissue samples histologically classified as normal and cancer were thawed, minced and fixed with formaldehyde. After glycine treatment and washes samples were treated with high energy ultrasound to shear the chromatin. The lysates were cleared by centrifugation, aliquoted and stored (-80°C). For MeDIP the input was deproteinized, RNase-treated and denatured DNA. MeDIP was done using anti-5mC antibody (Aviva) and ChIP assays with anti-Pol II CTD, H3K9/14Ac, H2A.Z and H3K27m3 antibodies. MeDIP and ChIP DNA were analyzed at the indicated site of the *SPARC *gene in real-time PCR. Data represent values for each individual sample (five normal and five cancer), expressed as % input.

**Figure 5:**
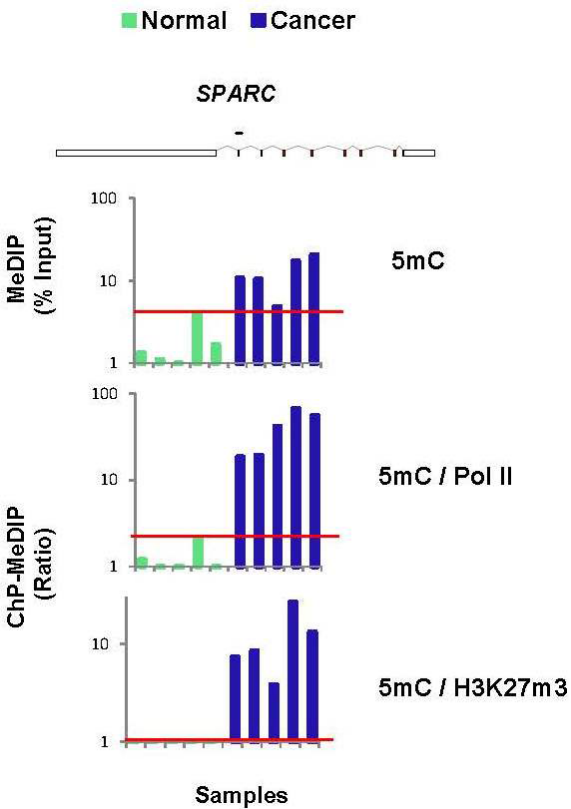
**Combined ChIP-MeDIP analysis at a single locus is sufficient to demarcate normal cervical tissues from cervical cancer**. 5mC data for five normal and five cancer samples is compared (Figure 4) to either 5mC/Pol II or 5mC/H3K27m3 ratio. The red horizontal line demarcates the highest value for the normal tissues.

Using MethyLight we have previously identified several genes that are methylated in cervical cancer, including *SPARC *[43]. We tested five normal human cervical tissues and five cervical cancer specimen using matrix ChIP-MeDIP assays (Figure 4). In agreement with our previous results [43] most of the cancer samples had higher levels of *SPARC *methylation assayed using either MethylLight (*row 1*) or MeDIP (*row 2*), suggesting that the gene is silenced in cervical cancer. Consistent with this suggestion *SPARC *Pol II levels (*row 3*) and histone marks levels (*rows 4-6*) were lower in cancer samples. For most of the samples there was a clear difference in *SPARC *methylation and histone marks in cancer compared to normal tissues. However the demarcation of cancer versus normal tissue was greater when DNA methylation was calculated as either Pol II or a histone mark ratio (Figure 5). Although this is a small pilot cohort, this simple example of an integrative analysis suggests that Matrix ChIP-MeDIP could be a more specific and sensitive method to differentiate tumor from normal tissues and that as few as one gene could be sufficient to make the distinction. The capability of the Matrix ChIP-MeDIP platform to assay DNA methylation and histone marks along with Pol II and enzyme recruitment to cancer-critical genes provides an avenue for more extensive integrative analysis to develop combinatorial biomarker panels to better characterize tumors diagnostically, prognostically and therapeutically. In this regard, cancer genome-wide chromatin studies and database resources could be used to exploit the Matrix ChIP-MeDIP platform.

### Application of Matrix ChIP-MeDIP to animal models of diabetic complication: Combined ChIP-MeDIP reveals diabetes-induced reciprocal changes in the levels of DNA methylation and Pol II at an inflammatory gene in diabetic kidney

Diabetic kidney disease, or diabetic nephropathy, is a major cause of kidney failure world-wide [53]. Chromatin biology of diabetic complications is merely a nascent field and only a very few studies examined chromatin changes in diabetic kidneys [54-56]. The progress in this area has been hampered by the lack of sufficiently sensitive methods to measure renal chromatin changes at specific gene loci in a diabetic milieu. Low grade inflammation triggered by the components of diabetic milieu is one of the contributors to this disease. In diabetic kidney disease there is early increase in the expression of the monocyte chemoattractant protein 1 (MCP-1) which promotes inflammation, kidney injury and fibrosis [57]. Later in the course of the disease there is enhanced expression of the transforming growth factor β (TGF-β1), which may result, in part, from the increased production of MCP-1 [58]. Both the *MCP-1 *and the *TGF-β1 *genes have CpG located in the vicinity of transcription start site.

The OVE26 mice on FVB background overexpress calmodulin gene in pancreatic β cells, resulting in hyperglycemia and early onset of type 1 diabetes and diabetic nephropathy [44,53]. The leptin deficient insulin resistant C57BL/6J ob/ob mice are mildly hyperglycemic and do not develop kidney lesions resembling human diabetes [59]. We used chromatin from the kidneys of these strains and their respective controls in the combined ChIP-MeDIP assay. Blood glucose levels in these strains were as follows (in mg/dl): C57BL/6J 65 ± 4; C57BL/6 ob/ob 112 ± 13; FVB 197 ± 15; and FVB OVE26 649 ± 22.

**Figure 6:**
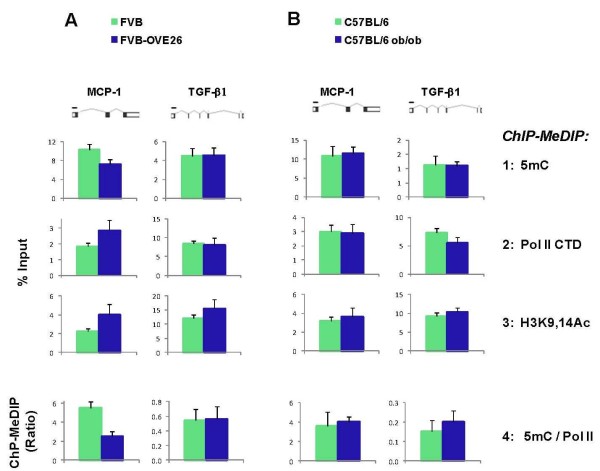
**Combined ChIP-MeDIP analysis reveals changes in methylation and Pol II levels at a pro-inflammatory gene locus in diabetic mouse kidney**. Sheared cross-linked kidney chromatin from two-month-old diabetic OVE26 (on FVB background) (***A***), and two-month-old leptin resistant insulin resistant obese C57BL/6J ob/ob mice (***B***) and their respective normal age-matched controls were assayed using anti-5mC, Pol II CTD and H3K9,14Ac antibodies. The same cross-linked chromatin samples were used for both MeDIP and ChIP assays. MeDIP and ChIP DNA were analyzed at the indicated sites of the *MCP-1 *and *TGF-β1 *genes in real-time PCR. Data represent mean ± SEM (6 animals from each strain), expressed either as % Input (*panels 1-3*) or 5mC/Pol II ratio (*panel 4*).

Matrix ChIP-MeDIP analysis revealed lower 5mC levels at the *MCP-1 *gene in the diabetic OVE26 mice compared to the normal FVB controls (Figure 6A*, row 1*). The lower DNA methylation levels in the diabetic mice were associated with higher levels of Pol II (*row 2*) as well as higher levels of H3K9,14Ac (*row 3*). To quantitate the relationship between DNA methylation and transcription we calculated the ratio of 5mC levels to Pol II density in each one of the individual kidney samples. The average 5mC/Pol II ratio at the *MCP-1 *gene in the OVE26 diabetic kidneys was less than 50% of that calculated in the normal FVB kidney. This analysis suggests that diabetes induces reciprocal changes in DNA methylation and Pol II transcription at the renal *MCP-1 *gene. To our knowledge this is the first demonstration that diabetic milieu alters DNA methylation at a specific gene locus in the kidney, an effect that may be contributing to increased transcription of the cognate gene. In contrast to the *MCP-1 *gene, there were no differences detected at the *TGF-β1 *locus. This suggests that early diabetes-induced chromatin changes are gene selective. Interestingly, we did not detect any differences between the lean and obese C57BL/6J strains in the kidney at either the *MCP-1 *or *TGF-β1 *gene (Figure 6B). Taken together, these results show that the combined Matrix ChIP-MeDIP platform can be applied to the analysis of chromatin and transcription processes in chronic kidney disease. Moreover, as in the case of cancer (Figure 5) the ratio of 5mC/Pol II at a relevant gene(s) may serve as a potential biomarker for kidney disease using either renal biopsy specimens or the hundreds of renal cells that are normally shed in urine every day [60].

Although compared to antibody-coated beads, the microplate ChIP and MeDIP offers higher throughput and better performance using 5mC (Figure 3B) and other selected antibodies [47] there are potential limitations that remain to be tested and solved. In the microplate assay the detection at selected genomic sites is in the range 0.5-50% of 100-200 ng input DNA/well [33,36,47]. Thus, for most of the antibodies tested the quantity of ChIP DNA output is in the range of few nanograms or less, limiting the number of genomic sites that can be probed by real-time PCR. In contrast to antibody-coated wells, the volume of antibody-coated beads in a single pull-down can be easily scaled up to increase the DNA yield to the range of 500-1000 ng needed in either microarrays or sequencing platforms for genome-wide ChIP or MeDIP studies. Thus, for most antibodies, including that to 5mC, the current microplate format remains to be adapted for genome-wide studies which may require the addition of whole genome amplification [61].

The microwell platform can be used to probe a wide range of genome-associated epitopes using either polyclonal or monoclonal antibodies [33,36,47,62]. Notably, with some antibodies the microplate performs better than the beads (Figure 3B and [47]). Given that there could be cases when genome-associated epitopes are not well detected by ChIP antibody-coated microplates, when necessary, the beads assay ought to be considered as an alternative for that specific epitope(s).

## Conclusions

We have developed a microplate-based MeDIP method that has several important advantages over beads assays. i) It simplifies the entire procedure eliminating the need for centrifugation or magnets and uses fewer buffers. ii) It is more sensitive and has a lower level of non-specific binding compared to beads. iii) The microplate ChIP platform is sufficiently sensitive to probe genome-associated enzymes such as kinases and phosphatases [33,36]. Thus, the capability to simultaneously assay DNA methylation, histone marks and signal transducers on the same plate(s) should facilitate mapping genomic pathways at selected genes. iv) The combined microplate ChIP-MeDIP and real-time PCR assays allows one to generate hundreds of ChIP-MeDIP records at a time to profile complex genomic events at selected disease-related gene loci. v) As the cost of genome-wide sequencing continues to decrease and user-friendly software tools becomes available, the Matrix ChIP-MeDIP output of hundreds of DNA samples could be potentially adapted to genome-wide studies and be generally affordable. Finally, the recent advances in DNA sequencing are generating extraordinary volumes of genome-wide chromatin data. Still, most of these studies have been done in cell cultures and largely lack mechanistic and functional information [63]. These valuable data resources are freely available providing great opportunities to integrate information across the different cell types and species allowing one to formulate mechanistic and functional hypothesis. The capability of Matrix ChIP-MeDIP to simultaneously study Pol II, histones, gene-associated transducers/enzymes and DNA methylation provides an unprecedented opportunity to better define the dynamic chromatin and transcription at specific gene loci.

## Competing interests

The authors declare that they have no competing interests.

## Authors' contributions

JY carried out the experiments, participated in design of the study and drafting the manuscript, QF prepared the cervical tissues and participated in the study design and drafting of the manuscript, YR and NK participated in the study design and drafting the manuscript, RK prepared the mouse kidney tissues and participated in drafting the manuscript, KB conceived of the idea, designed the study and wrote the manuscript. All authors read and approved the final manuscript

## References

[B1] Sharma S Kelly TK Jones PA Epigenetics in cancer Carcinogenesis 2010 31 27 36 10.1093/carcin/bgp220 PMC2802667 19752007

[B2] Heiser LM Wang NJ Talcott CL Laderoute KR Knapp M Guan Y Hu Z Ziyad S Weber BL Laquerre S Integrated analysis of breast cancer cell lines reveals unique signaling pathways Genome Biol 2009 10 R31 10.1186/gb-2009-10-3-r31 PMC2691002 19317917

[B3] Lund AH van Lohuizen M Epigenetics and cancer Genes Dev 2004 18 2315 2335 10.1101/gad.1232504 15466484

[B4] Villeneuve LM Natarajan R The role of epigenetics in the pathology of diabetic complications Am J Physiol Renal Physiol 2010 299 F14 25 10.1152/ajprenal.00200.2010 PMC2904177 20462972

[B5] El-Osta A Brasacchio D Yao D Pocai A Jones PL Roeder RG Cooper ME Brownlee M Transient high glucose causes persistent epigenetic changes and altered gene expression during subsequent normoglycemia J Exp Med 2008 205 2409 2417 10.1084/jem.20081188 PMC2556800 18809715

[B6] Hang CT Yang J Han P Cheng HL Shang C Ashley E Zhou B Chang CP Chromatin regulation by Brg1 underlies heart muscle development and disease Nature 2010 466 62 67 10.1038/nature09130 PMC2898892 20596014

[B7] Fraga MF Esteller M Epigenetics and aging: the targets and the marks Trends Genet 2007 23 413 418 10.1016/j.tig.2007.05.008 17559965

[B8] Li B Carey M Workman JL The role of chromatin during transcription Cell 2007 128 707 719 10.1016/j.cell.2007.01.015 17320508

[B9] Kouzarides T Chromatin modifications and their function Cell 2007 128 693 705 10.1016/j.cell.2007.02.005 17320507

[B10] Lister R Pelizzola M Dowen RH Hawkins RD Hon G Tonti-Filippini J Nery JR Lee L Ye Z Ngo QM Human DNA methylomes at base resolution show widespread epigenomic differences Nature 2009 462 315 322 10.1038/nature08514 PMC2857523 19829295

[B11] Gronbaek K Hother C Jones PA Epigenetic changes in cancer Apmis 2007 115 1039 1059 10.1111/j.1600-0463.2007.apm_636.xml.x 18042143

[B12] Laird PW The power and the promise of DNA methylation markers Nat Rev Cancer 2003 3 253 266 10.1038/nrc1045 12671664

[B13] Patra SK Patra A Rizzi F Ghosh TC Bettuzzi S Demethylation of (Cytosine-5-C-methyl) DNA and regulation of transcription in the epigenetic pathways of cancer development Cancer Metastasis Rev 2008 27 315 334 10.1007/s10555-008-9118-y 18246412

[B14] Choi JK Bae JB Lyu J Kim TY Kim YJ Nucleosome deposition and DNA methylation at coding region boundaries Genome Biol 2009 10 R89 10.1186/gb-2009-10-9-r89 PMC2768978 19723310

[B15] Choi JK Contrasting chromatin organization of CpG islands and exons in the human genome Genome Biol 2010 11 R70 10.1186/gb-2010-11-7-r70 PMC2926781 20602769

[B16] Bender CM Gonzalgo ML Gonzales FA Nguyen CT Robertson KD Jones PA Roles of cell division and gene transcription in the methylation of CpG islands Mol Cell Biol 1999 19 6690 6698 10.1128/mcb.19.10.6690 PMC84656 10490608

[B17] Pomraning KR Smith KM Freitag M Genome-wide high throughput analysis of DNA methylation in eukaryotes Methods 2009 47 142 150 10.1016/j.ymeth.2008.09.022 18950712

[B18] Sorensen AL Collas P Immunoprecipitation of methylated DNA Methods Mol Biol 2009 567 249 262 10.1007/978-1-60327-414-2_16 19588097

[B19] Eads CA Danenberg KD Kawakami K Saltz LB Blake C Shibata D Danenberg PV Laird PW MethyLight: a high-throughput assay to measure DNA methylation Nucleic Acids Res 2000 28 E32 10.1093/nar/28.8.e32 PMC102836 10734209

[B20] Ushijima T Morimura K Hosoya Y Okonogi H Tatematsu M Sugimura T Nagao M Establishment of methylation-sensitive-representational difference analysis and isolation of hypo- and hypermethylated genomic fragments in mouse liver tumors Proc Natl Acad Sci USA 1997 94 2284 2289 10.1073/pnas.94.6.2284 PMC20079 9122186

[B21] Jacinto FV Ballestar E Esteller M Methyl-DNA immunoprecipitation (MeDIP): Hunting down the DNA methylome Biotechniques 2008 44 35 42 10.2144/000112708 18254377

[B22] Wysocka J Swigut T Milne TA Dou Y Zhang X Burlingame AL Roeder RG Brivanlou AH Allis CD WDR5 Associates with Histone H3 Methylated at K4 and Is Essential for H3 K4 Methylation and Vertebrate Development Cell 2005 121 859 872 10.1016/j.cell.2005.03.036 15960974

[B23] Fischle W Tseng BS Dormann HL Ueberheide BM Garcia BA Shabanowitz J Hunt DF Funabiki H Allis CD Regulation of HP1-chromatin binding by histone H3 methylation and phosphorylation Nature 2005 438 1116 1122 10.1038/nature04219 16222246

[B24] Bernstein BE Meissner A Lander ES The mammalian epigenome Cell 2007 128 669 681 10.1016/j.cell.2007.01.033 17320505

[B25] Garcia BA Shabanowitz J Hunt DF Characterization of histones and their post-translational modifications by mass spectrometry Curr Opin Chem Biol 2007 11 66 73 10.1016/j.cbpa.2006.11.022 17157550

[B26] Trojer P Reinberg D Histone lysine demethylases and their impact on epigenetics Cell 2006 125 213 217 10.1016/j.cell.2006.04.003 16630806

[B27] Clayton AL Hazzalin CA Mahadevan LC Enhanced histone acetylation and transcription: a dynamic perspective Mol Cell 2006 23 289 296 10.1016/j.molcel.2006.06.017 16885019

[B28] Thiagalingam S Cheng KH Lee HJ Mineva N Thiagalingam A Ponte JF Histone deacetylases: unique players in shaping the epigenetic histone code Ann N Y Acad Sci 2003 983 84 100 10.1111/j.1749-6632.2003.tb05964.x 12724214

[B29] Hirota T Lipp JJ Toh BH Peters JM Histone H3 serine 10 phosphorylation by Aurora B causes HP1 dissociation from heterochromatin Nature 2005 438 1176 1180 10.1038/nature04254 16222244

[B30] He Z Cho YY Ma WY Choi HS Bode AM Dong Z Regulation of ultraviolet B-induced phosphorylation of histone H3 at serine 10 by Fyn kinase J Biol Chem 2005 280 2446 2454 10.1074/jbc.M402053200 15537652

[B31] Bennett D Transcriptional control by chromosome-associated protein phosphatase-1 Biochem Soc Trans 2005 33 1444 1446 10.1042/BST0331444 16246142

[B32] Edmunds JW Mahadevan LC Cell signaling. Protein kinases seek close encounters with active genes Science 2006 313 449 451 10.1126/science.1131158 16873633

[B33] Mikula M Bomsztyk K Direct recruitment of ERK cascade components to inducible genes is regulated by the heterogeneous nuclear ribonucleoprotein (HnRNP) K J Biol Chem 2011 286 9763 9775 10.1074/jbc.M110.213330 PMC3058954 21233203

[B34] Pokholok DK Zeitlinger J Hannett NM Reynolds DB Young RA Activated signal transduction kinases frequently occupy target genes Science 2006 313 533 536 10.1126/science.1127677 16873666

[B35] Bungard D Fuerth BJ Zeng PY Faubert B Mass NL Viollet B Carling D Thompson CB Jones RG Berger SL Signaling Kinase AMPK Activates Stress-Promoted Transcription via Histone H2B Phosphorylation Science 2010 329 1201 1205 10.1126/science.1191241 PMC3922052 20647423

[B36] Nelson JD Leboeuf RC Bomsztyk K Direct recruitment of insulin receptor and ERK signaling cascade to insulin-inducible gene loci Diabetes 2011 60 127 137 10.2337/db09-1806 PMC3012164 20929976

[B37] Trinkle-Mulcahy L Andersen J Lam YW Moorhead G Mann M Lamond AI Repo-Man recruits PP1 gamma to chromatin and is essential for cell viability J Cell Biol 2006 172 679 692 10.1083/jcb.200508154 PMC2063701 16492807

[B38] Moorhead GB Trinkle-Mulcahy L Ulke-Lemee A Emerging roles of nuclear protein phosphatases Nat Rev Mol Cell Biol 2007 8 234 244 10.1038/nrm2126 17318227

[B39] Orlando V Strutt H Paro R Analysis of chromatin structure by in vivo formaldehyde cross-linking Methods 1997 11 205 214 10.1006/meth.1996.0407 8993033

[B40] Solomon MJ Varshavsky A Formaldehyde-mediated DNA-protein crosslinking: a probe for in vivo chromatin structures Proc Natl Acad Sci USA 1985 82 6470 6474 10.1073/pnas.82.19.6470 PMC390738 2995966

[B41] O'Neill LP Turner BM Immunoprecipitation of chromatin Methods Enzymol 1996 274 189 197 10.1016/s0076-6879(96)74017-x 8902805

[B42] Cedar H Bergman Y Linking DNA methylation and histone modification: patterns and paradigms Nat Rev Genet 2009 10 295 304 10.1038/nrg2540 19308066

[B43] Sova P Feng Q Geiss G Wood T Strauss R Rudolf V Lieber A Kiviat N Discovery of novel methylation biomarkers in cervical carcinoma by global demethylation and microarray analysis Cancer Epidemiol Biomarkers Prev 2006 15 114 123 10.1158/1055-9965.EPI-05-0323 16434596

[B44] Zheng S Noonan WT Metreveli NS Coventry S Kralik PM Carlson EC Epstein PN Development of late-stage diabetic nephropathy in OVE26 diabetic mice Diabetes 2004 53 3248 3257 10.2337/diabetes.53.12.3248 15561957

[B45] Gletsu NA Field CJ Clandinin MT Obese mice have higher insulin receptor levels in the hepatocyte cell nucleus following insulin stimulation in vivo with an oral glucose meal Biochim Biophys Acta 1999 1454 251 260 10.1016/s0925-4439(99)00043-5 10452959

[B46] Nelson JD Denisenko O Bomsztyk K Protocol for the fast chromatin immunoprecipitation (ChIP) method Nat Protoc 2006 1 179 185 10.1038/nprot.2006.27 17406230

[B47] Flanagin S Nelson JD Castner DG Denisenko O Bomsztyk K Microplate-based chromatin immunoprecipitation method, Matrix ChIP: a platform to study signaling of complex genomic events Nucleic Acids Res 2008 36 e17 10.1093/nar/gkn001 PMC2241906 18203739

[B48] Nelson J Denisenko O Bomsztyk K Profiling RNA polymerase II using the fast chromatin immunoprecipitation method Methods Mol Biol 2011 703 219 234 10.1007/978-1-59745-248-9_15 21125493

[B49] Feng Q Hawes SE Stern JE Wiens L Lu H Dong ZM Jordan CD Kiviat NB Vesselle H DNA methylation in tumor and matched normal tissues from non-small cell lung cancer patients Cancer Epidemiol Biomarkers Prev 2008 17 645 654 10.1158/1055-9965.EPI-07-2518 PMC2798850 18349282

[B50] Yang AS Estecio MR Doshi K Kondo Y Tajara EH Issa JP A simple method for estimating global DNA methylation using bisulfite PCR of repetitive DNA elements Nucleic Acids Res 2004 32 e38 10.1093/nar/gnh032 PMC373427 14973332

[B51] Feng Q Stern JE Hawes SE Lu H Jiang M Kiviat NB DNA methylation changes in normal liver tissues and hepatocellular carcinoma with different viral infection Exp Mol Pathol 2010 88 287 292 10.1016/j.yexmp.2010.01.002 PMC2848881 20079733

[B52] Hanahan D Weinberg RA Hallmarks of cancer: the next generation Cell 2011 144 646 674 10.1016/j.cell.2011.02.013 21376230

[B53] Brosius FC Alpers CE Bottinger EP Breyer MD Coffman TM Gurley SB Harris RC Kakoki M Kretzler M Leiter EH Mouse models of diabetic nephropathy J Am Soc Nephrol 2009 20 2503 2512 10.1681/ASN.2009070721 PMC4075053 19729434

[B54] Sun G Reddy MA Yuan H Lanting L Kato M Natarajan R Epigenetic histone methylation modulates fibrotic gene expression J Am Soc Nephrol 2010 21 2069 2080 10.1681/ASN.2010060633 PMC3014020 20930066

[B55] Sayyed SG Gaikwad AB Lichtnekert J Kulkarni O Eulberg D Klussmann S Tikoo K Anders HJ Progressive glomerulosclerosis in type 2 diabetes is associated with renal histone H3K9 and H3K23 acetylation, H3K4 dimethylation and phosphorylation at serine 10 Nephrol Dial Transplant 2010 25 1811 1817 10.1093/ndt/gfp730 20067909

[B56] Villeneuve LM Reddy MA Natarajan R Epigenetics: deciphering its role in diabetes and its chronic complications Clin Exp Pharmacol Physiol 2011 38 401 409 10.1111/j.1440-1681.2011.05497.x PMC3123432 21309809

[B57] Tesch GH MCP-1/CCL2: a new diagnostic marker and therapeutic target for progressive renal injury in diabetic nephropathy Am J Physiol Renal Physiol 2008 294 F697 701 10.1152/ajprenal.00016.2008 18272603

[B58] Kanamori H Matsubara T Mima A Sumi E Nagai K Takahashi T Abe H Iehara N Fukatsu A Okamoto H Inhibition of MCP-1/CCR2 pathway ameliorates the development of diabetic nephropathy Biochem Biophys Res Commun 2007 360 772 777 10.1016/j.bbrc.2007.06.148 17631861

[B59] Hudkins KL Pichaiwong W Wietecha T Kowalewska J Banas MC Spencer MW Muhlfeld A Koelling M Pippin JW Shankland SJ BTBR Ob/Ob mutant mice model progressive diabetic nephropathy J Am Soc Nephrol 2010 21 1533 1542 10.1681/ASN.2009121290 PMC3013527 20634301

[B60] Vogelmann SU Nelson WJ Myers BD Lemley KV Urinary excretion of viable podocytes in health and renal disease Am J Physiol Renal Physiol 2003 285 F40 48 10.1152/ajprenal.00404.2002 PMC3368602 12631553

[B61] Lao K Xu NL Straus NA Whole genome amplification using single-primer PCR Biotechnol J 2008 3 378 382 10.1002/biot.200700253 18293327

[B62] Aker M Bomsztyk K Emery DW POLY(ADP-RIBOSE) polymerase-1 (Parp-1) contributes to the barrier function of a vertebrate chromatin insulator J Biol Chem 2010 285 37589 37597 10.1074/jbc.M110.174532 PMC2988364 20876582

[B63] Ernst J Kheradpour P Mikkelsen TS Shoresh N Ward LD Epstein CB Zhang X Wang L Issner R Coyne M Mapping and analysis of chromatin state dynamics in nine human cell types Nature 2011 473 43 49 10.1038/nature09906 PMC3088773 21441907

